# Effects of *Hibiscus sabdariffa* Calyxes Aqueous Extract on Antioxidant Status and Histopathology in Mammary Tumor-Induced in Rats

**DOI:** 10.1155/2022/9872788

**Published:** 2022-04-13

**Authors:** Thierry Renaud Bassong, Larissa Vanelle Kenmogne, Charline Florence Awounfack, Derek Tantoh Ndinteh, Dieudonné Njamen, Stéphane Zingue

**Affiliations:** ^1^Department of Animal Biology and Physiology, Faculty of Science, University of Yaoundé 1, P.O. Box 812, Yaounde, Cameroon; ^2^Department of Surgery, Faculty of Health Sciences, University of the Witwatersrand, Johannesburg 2193, South Africa; ^3^Centre for Natural Product Research, Department of Chemical Sciences, University of Johannesburg, P.O. Box 17011, Doornfontein, Johannesburg 2028, South Africa; ^4^Department of Medical and Biomedical Engineering, Higher Technical Teachers' Training College, University of Ebolowa, P.O. Box 886 Ebolowa, Cameroon

## Abstract

Breast cancer is a major threat worldwide. *Hibiscus sabdariffa* is widely consumed as beverage in sub-Saharan Africa for its anticancer potential. The present study therefore aimed at scientifically verifying its anticancer effect in rats. For this, 48 Wistar rats (∼55 days) were treated either with tamoxifen at 3.3 mg/kg BW (standard) or with a decoction of *H*. *sabdariffa* (125, 250, and 500 mg/kg BW) or distilled water (vehicle). Breast cancer was induced by a single dose of 50 mg/kg of 7,12-dimethylbenz(a)anthracene (DMBA). At the end of the 21 weeks of treatment, the tumor incidence, tumor morphology, histopathology, as well as some biochemical parameters in the tumors were assessed. As a result, 86% of DMBA's rats developed mammary tumors. The *H*. *sabdariffa* extract (125 and 250 mg/kg) reduced tumor incidence by 63% and 75%, respectively; inhibited tumor burden by 84.86% and 38.78%, respectively, and decreased tumor volume by more than 72% compared to the DMBA group. It also protected rats against DMBA-induced diffuse breast neoplasia, and the optimal effect was recorded at 125 mg/kg. Furthermore, it significantly increases the SOD activity and decreases the MDA level. In summary, *H. sabdariffa* has antibreast tumor and antioxidant properties in rats, which could justify its common use to treat cancer.

## 1. Introduction

*Cancer* is nowadays a major public health problem in many parts of the world [1]. It is a group of diseases characterized by an uncontrolled multiplication of cells that can move and cause damage in other systems [2]. *Cancer* remains one of the leading causes of death worldwide with 10 million deaths out of 19.3 million cases in 2020 [3]. It is expected ∼28.4 million cases by 2040 if nothing is done; a 47% rise from 2020 [3]. With 11.7% of new cases, breast cancer is the most diagnosed cancer over the world and it affects younger women [4]. Breast cancer has a multifactorial etiology which may include but is not limited to sex, age, prolonged exposure of the mammary gland to endogenous and exogenous estrogens, genetic factors (mutations in suppressor genes BRCA1 and BRCA2 tumors), and exposure to polycyclic aromatic hydrocarbons (e.g. DMBA) [5, 6].

In Cameroon, it represents 20% of all cancers in women with ∼4,000 new cases out of which ∼2000 cases die [7,8]. Just like in other developing countries, in Cameroon, patients present when the cancer is already at an advanced stage. This delay in diagnosis is mainly attributed to the low level of knowledge of patients and a limited health system. The treatment of breast cancer has improved in recent decades, and several methods (surgery, hormone therapy, chemotherapy, radiation therapy, and immunotherapy) are used to combat this disease. However, its high cost, adverse effects, and systemic toxicity of current chemotherapeutic as well as molecules drug resistance constitute the main limitations to treatment [9]. As an alternative, many patients therefore rely to natural substances, which have acceptable toxicity and potential to manage cancer and pain [10]. Therefore, the discovery of new chemopreventive substances is an urgent need since 70% of cancer deaths are recorded in developing countries [11].

To contribute to this search, we investigated a very popular beverage in sub-Saharan Africa made from “bissap” (*Hibiscus sabdariffa*) to prevent cancer. It is a plant belonging to the Malvaceae family mainly found in Equatorial Guinea, Ghana, Congo, and Cameroon [12]. Exploited to concoct in some of these countries delicious dishes or drinks, this plant stood out from others for its numerous virtues such as analgesic, antidiarrhoeal, anti-inflammatory, antihypertensive, antibacterial, and anticancer properties [13]. *Hibiscus sabdariffa* extracts and its isolates were reported to induce selective cytotoxicity, cell cycle arrest, apoptosis, and chemoprevention in cell and animal models. Moreover, it has a high amount of phenolic compounds; out of them, delphinidin-3-sambubioside, quecertin, and protocatechuic acid have been demonstrated to be potent anticancer agents [14]. To the best of our knowledge, no *in vivo* study has yet been conducted on the potential of “Bissap” to prevent breast cancer. Therefore, this study was aimed at assessing the anticancer effects of the decoction of *Hibiscus sabdariffa* on antioxidative status, breast cancer biomarker CA 15-3, total proteins, hematology, and histopathology of the mammary glands in DMBA-induced breast cancer in rats.

## 2. Materials and Methods

### 2.1. Chemicals and Reagents

The chemical carcinogen 7,12-dimethylbenz(a)anthracene (DMBA, purity ≥95%) was acquired from Sigma-Aldrich (Stanford, Germany). The standard drug used against estrogen-dependent breast cancers tamoxifen citrate (Mylan®) was obtained from MYLAN SAS (Saint-Priest, France). The anesthetics such as diazepam (Valium® 10 mg/2 mL) and ketamine (Ketamine hypochloride 50 mg/mL) were obtained from Roche (Fontenay-sous-Bois, France) and Rotex Medica (Tritau, Germany), respectively. *Cancer* antigen 15-3 (CA 15-3) enzyme-linked immunosorbent assay (ELISA) kit was from Monobind Inc® (California, USA). All biochemical reagent kits were from Fortress Diagnostics Limited® (Muckamore, United Kingdom).

### 2.2. Plant Collection and Authentication

The calyxes of *Hibiscus sabdariffa* flowers were ordered from a wholesaler in the city of Yaoundé (Centre Region, Cameroon). The sample used was authenticated at the National Herbarium of Cameroon (HNC) by comparison with Westphal material N° 9970 of Herbarium collection specimen N° 42822.

### 2.3. Decoction of *Hibiscus* sabdariffa Calyxes

The procedure of artisanal preparation of “bissap” juice as done in Cameroon was applied. In fact, the calyxes of *Hibiscus sabdariffa* were dried in the shade for a day before being sorted. Subsequently, 300 g of calyxes were mixed with 3 liters of tap water and boiled for 1 hour 30 minutes. The obtained mixture was filtered using a Wattman filter paper N°4 and dried in a ventilation oven (50°C). This process was repeated thrice and afforded 54 g of crude aqueous extract of *Hibiscus sabdariffa*.

### 2.4. Phytochemical Characterization of *Hibiscus* sabdariffa Extract

#### 2.4.1. Total Proteins Content

The total protein content in the extract was determined by the method of Bradford [15], using bovine serum albumin (BSA) as standard. Technically, 1 mL of extract was added to same volume of freshly prepared Bradford reagent. Absorbance was read at 595 nm using a UV-VIS 1605 Shimadzu spectrophotometer (Mettler Teledo®, New York, USA) after 30 min of incubation in darkness.

#### 2.4.2. Total Phenolic Compounds Content

The phenolic compounds were quantified in the extract using the Folin–Ciocalteu reagent as described by Chlopicka et al. [16]. For this, 75 *µ*L of Folin–Ciocalteu reagent was added to 750 *µ*L of extract (1 mg/mL). After 3 min, 750 *μ*L of Na_2_CO_3_ (20%) was added, followed by a 30 min incubation in darkness. The absorbance was measured at 760 nm using a UV-VIS 1605 Shimadzu spectrophotometer. In this experiment, ferulic acid served as standard.

#### 2.4.3. Total Flavonoids Content

The total flavonoids content was determined in this extract using the modified aluminum chloride colorimetric method of Chang et al. [17]. Hence, 0.5 mL of *Hibiscus sabdariffa* extract or distilled water (blank) or standard were introduced in dry tubes, and 0.1 mL of 10% aluminum chloride, 0.1 mL of 1 M potassium acetate,1.5 mL of 80% methanol, and 2.8 mL distilled water were added and mixed. The solutions were then incubated at room temperature for 30 min, and absorbance was read at 415 nm. The concentration of flavonoids was expressed as *µ*g quercetin equivalent (QE) per mg extract.

#### 2.4.4. Total Polysaccharides Content

The measurement of total polysaccharides content was performed using the colorimetric method of phenol-H_2_SO_4_, with glucose as standard. The neutral monosaccharides were heated in acid medium and transformed to dehydrated derivatives of furfural. In each tube containing 0.2 mL of extracts, 0.2 mL of 5% phenol and 1 mL of concentrated sulfuric acid were added. The mixtures were stirred and incubated at 100°C for 10 min, and the absorbance was read at 485 nm using a UV-VIS 1605 Shimadzu spectrophotometer. The amount of total polysaccharides was expressed as *μ*g equivalent glucose (EG)/mg of dry extract.

### 2.5. DMBA-Induced Breast Cancer in Rats

#### 2.5.1. Experimental Animals

Forty-eight (48) prepubertal Wistar rats aged 35 to 40 days at the start of the experiment and weighing ∼50 g were procured from the animal facility of the Laboratory of Animal Physiology (University of Yaoundé I, Cameroon). They were grouped and allowed at room temperature in prefabricated plastic cages. Throughout the experiment, the rats had free access to standard rat chow and water. The composition of animal diet was corn (36.7%), bone flour (14.5%), wheat (36.6%), fish flour (4.8%), crushed palm kernel (7.3%), sodium chloride (0.3%), and vitamin complex (Olivitazol®- 0.01%).

Animal handling and experimental procedures were conducted following the directives of the Institutional Ethics Committee of the Ministry of Scientific Research and Innovation of Cameroon, which adopted the directives established by the European Union on the care of animals (EEC Council 86/609).

#### 2.5.2. Determination of Doses of Different Substances

The carcinogen DMBA was given at a dose of 50 mg/kg [18] while the standard tamoxifen was given at a dose of 3.3 mg/kg [19]. The decoction of *Hibiscus sabdariffa* was administered at doses of 125, 250, and 500 mg/kg BW. These doses were calculated from an average intake of 0.5 L of artisanal “Bissap” juice per day for a 70 kg adult.

#### 2.5.3. Treatment of Rats

To evaluate the chemopreventive effects of *H*. *sabdariffa* extract in DMBA-induced breast carcinogenesis in rats, 48 female Wistar rats aged 35 to 40 days were allowed to acclimatize for 10 days. At ∼50 days old, they were randomized into six (6) groups of eight (8) animals each (*n* = 8). Group I served as normal control, group II as negative control, and both received distilled water (vehicle). Group III served as positive control and received tamoxifen at the dose of 3.3 mg/kg BW. Groups IV, V, and VI were treated with *H*. *sabdariffa* extract at doses of 125, 250, and 500 mg/kg BW, respectively. All treatments were by intragastric gavage ten days before induction of breast tumors, once daily at around 8 a.m, and continued for 21 weeks.

#### 2.5.4. Induction of Breast Cancer

Breast tumors were induced in the prepubertal rats of groups II to VI. Then, 50 mg/kg BW of DMBA dissolved in 1 mL of olive oil was homogenized in ultrasound (ElmasonicS100H) and injected subcutaneously in the right inguinal mammary gland of each rat [20]. Normal rats received olive oil instead.

#### 2.5.5. Anticancer Effects Evaluation

To assess weight gain and early detection of mammary tumors, animals were weighed every week and palpated twice a week. Moribund animals were immediately sacrificed, and autopsy was done on those that died during the experiment. All animals that survived were sacrificed at the end of the study period by decapitation under ketamine and diazepam anesthesia (10 mg/kg and 50 mg/kg B W. *i*.*p*., respectively) after 12 h nonhydric fasting. Blood samples were collected in anticoagulant ethylenediaminetetraacetic (EDTA) tubes for hematological analysis and in dry tubes and centrifuged at 600 × *g* for 15 min at 4°C for subsequent biochemical analyzes. Thereafter, tumors were removed, counted, and weighed. A 1-mm precision caliper (IGAGING®) was used to measure tumor size, and the formula of Faustino-Rocha et al. [21] (length × weight × height × *π*/6) was used to calculate tumor volume. Brain, liver, lungs, kidneys, femur, uterus, vagina, and mammary glands were removed, weighed, and fixed in 10% formalin for histological analysis. The relative organ weight (mg/kg) was calculated as the organ weight (mg)/body weight (kg).

The tumor incidence and tumor burden corresponded to the percentage of affected animals per group and the total relative mass of tumors in one group, respectively. The percentage of inhibition of the tumor burden was calculated as follows: % inhibition of the tumor burden = [(tumor burden in DMBA group - tumor burden in test groups)/tumor burden in DMBA] × 100.

#### 2.5.6. Preparation of Homogenates of Mammary Glands and Tumors

Part of the mammary glands and tumors were cut, weighed, and ground in sodium phosphate buffer (0.1 M; pH 7.5) using Potter Teflon-glass on ice to afford a 20% final homogenate. After centrifugation at 3000 rpm for 15 minutes at 4°C, the supernatant collected was stored at −20°C for the determination of the total protein level.

### 2.6. Hematological Analysis

The following hematological parameters: white blood cell count (WBC), % of lymphocytes, % of monocytes, % of granulocytes, red blood cell count (RBC), hematocrit, hemoglobin, mean corpuscular volume (MCV), mean corpuscular hemoglobin (MCH), mean corpuscular hemoglobin concentration (MCHC), and platelet count were measured using a MINDRAY BC-2800 Auto Hematology Analyzer (Shenzhen Mindray Bop-medical Electronics®, Shenzhen, China).

### 2.7. Analysis of Oxidative Stress Makers

For the estimation of some oxidative stress makers in the homogenates of mammary glands and tumors, superoxide dismutase (SOD) and catalase activities were measured following the methods of Misra [22] and Sinha [23], respectively. Lipid membrane peroxidation was assessed through the estimation of the malondialdehyde (MDA) level, as described by Wilbur et al. [24].

### 2.8. ELISA Determination of CA 15-3

The measurement of the breast cancer biomarker CA 15-3, which is considered as the first circulating prognostic factor for breast cancer, was measured in sera using Cell Biolabs' *Cancer* Antigen 15-3 ELISA kit (Monobind Inc®, California, USA). The CA 15-3 assay measures shed or soluble forms of mucin-1 (MUC-1) protein, which is a transmembrane protein consisting of 2 subunits forming a stable dimer expressed at the apical plasma membrane of epithelial cells. The kit has detection sensitivity limit of 4 U/mL CA 15-3. The assay was performed following the manufacturer's instructions.

### 2.9. Histological Analysis

Organs (mammary glands, brain, liver, kidney, and lung) and tumors were dehydrated by a series of ethanol solutions and embedded in paraffin blocks before cutting into 5 *μ*m sections and stained with hematoxylin and eosin. Histomorphological changes were determined under an Axioskop 40 microscope connected to a computer where the image was transferred using MRGrab1.0 and Axio Vision 3.1 softwares (Zeiss®, Hallbermoos, Germany).

### 2.10. Statistical Analysis

The data were expressed as mean ± standard error of mean. The effects of different treatment-based extracts were compared against those of the control using one-way analysis of variance (ANOVA) followed by Dunnett's post hoc test. All analyzes were performed with GraphPad Prism 5.0 (San Diego, CA, USA). *p* *<* *0*.*05* was considered statistically significant.

## 3. Results

### 3.1. Phytochemical Characterization

As presented in Table 1, proteins, phenolic compounds, flavonoids, and polysaccharides were present in the *H*. *sabdariffa* extract. The total protein content was 17.09 ± 3.07 *µ*g eq BSA/mg of dry weight, the phenolic compound content was 288.83 ± 0.5 *µ*g FAE/mg of dry weight, and the flavonoid content was 209.76 ± 0.99 *µ*g eq quercetin/mg in crude extract. Moreover, it contains 29.61 ± 0.03 *μ*g eq glucose/mg dry weight of polysaccharides.

### 3.2. Chemopreventive Effect of *H. sabdariffa* Extract on Breast Tumors

#### 3.2.1. Effects on the Incidence of Deaths and Body Weight

**Figure 1:**
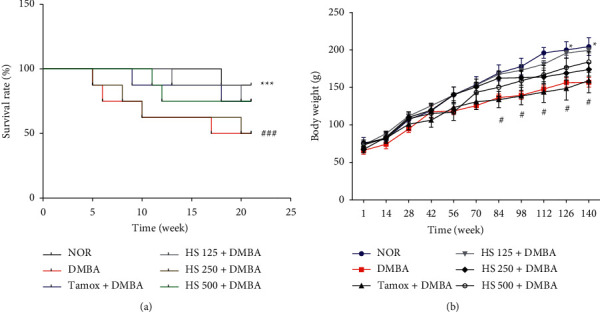
Kaplan–Meier curve (a) of the effects of *H*. *sabdariffa* extract on the survival of animals and body weight evolution (b) during 21 weeks post-DMBA induction. NOR = Animals serving as normal control and receiving distilled water (vehicle); DMBA = Animals serving as negative control and receiving distilled water (vehicle); TAMOX + DMBA = Animals serving as positive control and receiving tamoxifen at a dose of 3.3 mg/kg; and *H*. *sabdariffa* + DMBA = Animals receiving *H*. *sabdariffa* calyxes extract at doses of 125, 250, and 500 mg/kg. All animals except those in the normal group (NOR) were given a single dose of DMBA (50 mg/kg; *s*.*c*.). #*p* < 0.05, ### *p* < 0.001 compared to the normal control (NOR) and  ^*∗*^*p* < 0.05,  ^*∗∗∗*^*p* < 0.001 compared to the negative control.

During the 21 weeks of this experiment, at least one death was observed in each group (Figure 1(a)). The lowest and highest death rates were recorded in the normal group (NOR) [with a survival of 85% (1/8 rats)] and in the DMBA and *H*. *sabdariffa* 500 mg/kg groups [with a survival of 62.5% (e.g. 3/8 rats)]. Animals treated with tamoxifen and *H*. *sabdariffa* at 125 and 250 mg/kg BW showed a 75% survival rate (2/8 rats).

No great difference in body weights was observed during the 21 weeks of the study, except for rats in the DMBA and *H*. *sabdariffa* 125 mg/kg groups. A significant decrease (*p* < 0.05) in body weights of DMBA rats was observed from day 84 to the end of the experiment compared to the normal rats. Moreover, rats that received *H*. *sabdariffa* at 125 mg/kg had a significant increase (*p* < 0.05) in body weights from day 126 until the end of the experiment compared to the DMBA rats.

#### 3.2.2. Effect on Tumor Incidence and Tumor Burden

The results presented in Table 2 show the effect of *H*. *sabdariffa* extract on various tumor parameters. Thus, the tumor incidence was 86% in DMBA rats (7/8 rats). Tamoxifen treatment significantly (*p* < 0.001) protected rats against tumor incidence (50%) as well as the increased total tumor burden compared to the DMBA group. As for *H*. *sabdariffa* at doses 125 (*p* < 0.001) and 250 mg/kg BW (*p* < 0.01), there was significant decrease in the tumor burden compared to the DMBA group. A tumor incidence of 63% was recorded in rats treated with 125 or 250 mg/kg and 75% in those treated with 500 mg/kg BW of *H*. *sabdariffa*. The lowest doses of *H*. *sabdariffa* seemed more protective.

NOR = Animals serving as normal control and receiving distilled water (vehicle); DMBA = Animals serving as negative control and receiving distilled water (vehicle); TAMOX + DMBA = Animals serving as positive control and receiving tamoxifen at a dose of 3.3 mg/kg; and *H*. *sabdariffa* + DMBA = Animals receiving *H*. *sabdariffa* calyxes extract at doses of 125, 250, and 500 mg/kg. All animals except those in the normal group (NOR) were exposed to a single dose of DMBA (50 mg/kg; *s*.*c*.). ###*p* < 0.001 compared to the normal group (NOR); ^*∗∗∗*^*p* < 0.001 and ^*∗∗*^*p* < 0.01 compared to the DMBA group.

#### 3.2.3. Effect on Tumor Volume

**Figure 2:**
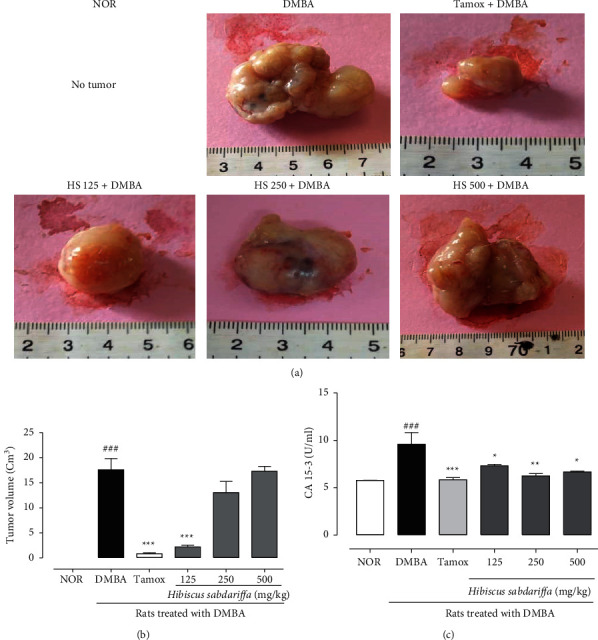
Effects of *H*. *sabdariffa* calyxes extract on tumor morphology (a), volume (b), and CA 15-3 levels (c). NOR = Animals serving as normal control and receiving distilled water (vehicle); DMBA = Animals serving as negative control and receiving distilled water (vehicle); TAMOX + DMBA = Animals serving as positive control and receiving tamoxifen at a dose of 3.3 mg/kg; and *H*. *sabdariffa* + DMBA = Animals receiving *H*. *sabdariffa* calyxes extract at doses of 125, 250, and 500 mg/kg. All animals except those in the normal group (NOR) were exposed to a single dose of DMBA (50 mg/kg; *s*.*c*.). ### *p* < 0.001 compared to the NOR group and ^∗^*p* < 0.05, ^∗∗^*p* < 0.01 ^∗∗∗^*p* < 0.001 compared to the DMBA group.

Figure 2(a) presents that the largest tumor was noticed in the DMBA group with an average tumor volume of 95.52 cm^3^. Tamoxifen inhibited tumor growth with an average tumor volume of 8.3 cm^3^ (*p* < 0.001) compared to DMBA rats. Animals treated with *H*. *sabdariffa* at 125 mg/kg significantly inhibited (*p* < 0.001) tumor growth with a volume of 22.99 cm^3^ compared to DMBA rats.

#### 3.2.4. Effect on the Breast Cancer Biomarker CA 15-3

Figure 2(b) shows the CA 15-3 levels in the DMBA group, which is significantly (*p* < 0.001) higher than that of the normal group. Animals that received either tamoxifen or H. sabdariffa extract doses tested, exhibited significant reduction (at least (*p* < 0.001), of this biomarker level as compared to the DMBA group.

#### 3.2.5. Effect on Antioxidant Status

The results presented in Table 3 show the effects of *H*. *sabdariffa* extract on some markers of oxidative stress in the homogenate of the mammary gland or mammary tumors after 21 weeks of treatment. There was a significant (*p* < 0.01) increase in superoxide dismutase (SOD) activity in rats treated with *H*. *sabdariffa* extract doses tested compared to the DMBA group. Animals treated with tamoxifen had an increment (*p* < 0.01) in SOD activity compared to the DMBA group.

Further, a significant increase (*p* < 0.001) in catalase activity was noted in rats of the DMBA group compared to normal rats. The tamoxifen-treated rats showed significantly (*p* < 0.001) lower activity than the DMBA group. Animals treated with *H*. *sabdariffa* extract exhibited a significant increase in catalase activity at 125 (*p* < 0.001), 250 (*p* < 0.01), and 500 (*p* < 0.01) mg/kg BW compared to the DMBA group.

Regarding the nitric oxide (NO) level, only the tamoxifen group significantly increased (*p* < 0.001) the level of NO in tumors compared to animals of the DMBA group. Although not significant, rats treated with *H*. *sabdariffa* extract at doses of 250 and 500 mg/kg BW showed a high level of NO compared to rats of the DMBA group.

NOR = Animals serving as normal control and receiving distilled water (vehicle); DMBA = Animals serving as negative control and receiving distilled water (vehicle); TAMOX + DMBA = Animals serving as positive control and receiving tamoxifen at a dose of 3.3 mg/kg; and *H*. *sabdariffa* + DMBA = Animals receiving *H*. *sabdariffa* calyxes extract at doses of 125, 250, and 500 mg/kg. All animals except those in the normal group (NOR) were exposed to a single dose of DMBA (50 mg/kg; *s*.*c*.). ###*p* < 0.001 compared to the normal group (NOR); ^∗^*p* < 0.05, ^∗∗^*p* < 0.01, and ^∗∗∗^*p* < 0.001 compared to the DMBA group.

No significant variation was recorded in the MDA level between the different groups, except for the group treated with *H*. *sabdariffa* extract at 125 mg/kg. However, a decrease of the MDA level was observed tested doses of *H*. *sabdariffa* extract compared to DMBA rats.

#### 3.2.6. Effect on Microarchitecture of Mammary Gland and Tumors

**Figure 3:**
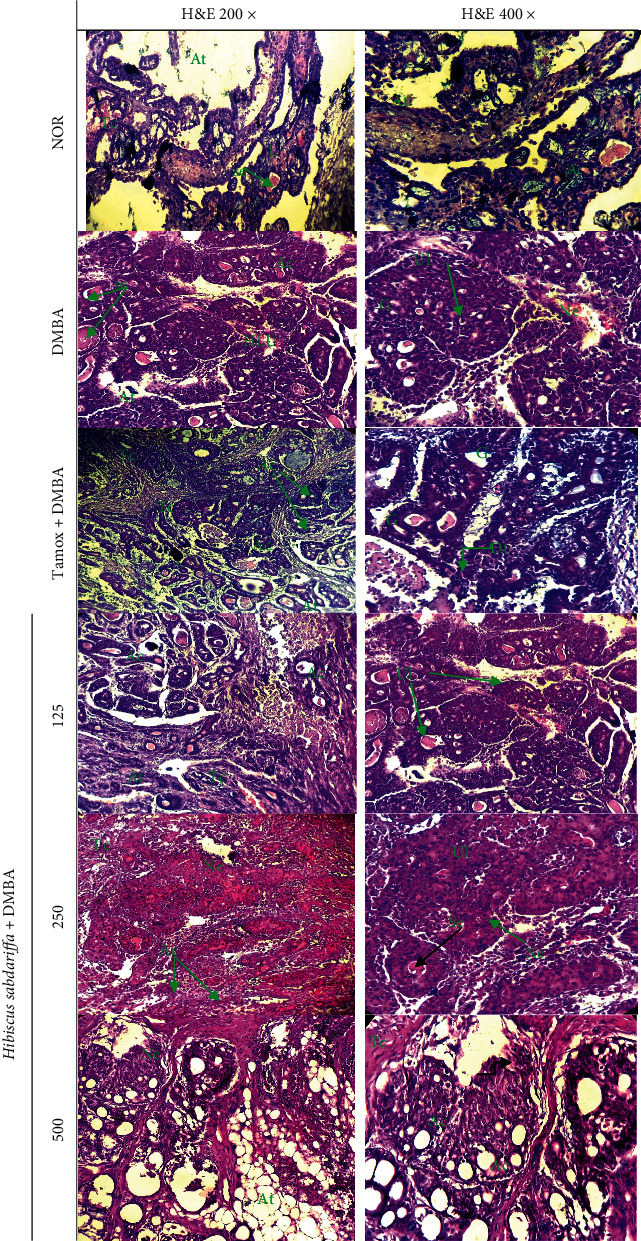
Photomicrographs (HE × 400) of cross-section of mammary gland (NOR) or tumors obtained after 21 weeks of treatment with different substances. NOR = Animals serving as normal control and receiving distilled water (vehicle); DMBA = Animals serving as negative control and receiving distilled water (vehicle); TAMOX + DMBA = Animals serving as positive control and receiving tamoxifen at a dose of 3.3 mg/kg; and *H*. *sabdariffa* + DMBA = Animals receiving *H*. *sabdariffa* calyxes extract at doses of 125, 250, and 500 mg/kg. All animals except those in the normal group (NOR) were exposed to a single dose of DMBA (50 mg/kg; *s*.*c*.). La = light of the acini; Ca = acinar cells; At = adipose tissue; Se = eosinophilic secretion, *L* = lobule; ULH = hypertrophied lobular unit Ne = neoplasia; Ul = ulcerative degradation.

At the end of the experiment, the histoarchitecture of the mammary gland and tumors following H&E staining is presented in Figure 3. Mammary glands of normal rats presented normal lobules with normal acini surrounded by a large amount of fatty tissue and a small amount of fibrous connective tissue. Histopathology of mammary tumors of rats in the DMBA group revealed cribriform adenocarcinoma of SBR grade III, characterized by accentuated hyperplasia with dilated ducts and a small amount of fatty tissue, disorganization of the breast parenchyma with abnormal cells. In opposite, tamoxifen-treated rats had for most of them a quasinormal histoarchitechture of mammary gland; however, some fibrosarcoma of SBR grade I was observed in this group. Analysis of mammary tumors from rats treated with the lowest dose of *H*. *Sabdariffa* extract revealed an adenocarcinoma of SBR grade I, evidenced by a weak disorganization of the mammary cells and parenchyma. However, rats treated with 250 or 500 mg/kg of *H*. *Sabdariffa*, presented adenocarcinoma of SBR grade II and III characterized by pronounced neoplasia, disorganization of parenchyma and abnormal cells.

### 3.3. Evaluation of Safety of the Extract during the Treatment

#### 3.3.1. Effect on Relative Organ Weights

DMBA significantly increased the liver (*p* < 0.01), spleen (*p* < 0.05), kidney (*p* < 0.05), femur (*p* < 0.05), and brain (*p* < 0.05) wet weights as compared to the normal group (Table 4). No significant variation was observed between *H*. *sabdariffa* treated groups and normal as well as DMBA groups, except for the dose of 125 mg/kg, which significantly (*p* < 0.05) decreased the lung wet weight compared to the DMBA group. A significant increase in femur (*p* < 0.01) and brain (*p* < 0.05) wet weights was observed in tamoxifen-treated rats compared to the normal group. In addition, it exhibited a significant decrease (*p* < 0.05) in the uterus wet weight compared to the normal group.

NOR = Animals serving as normal control and receiving distilled water (vehicle); DMBA = Animals serving as negative control and receiving distilled water (vehicle); TAMOX + DMBA = Animals serving as positive control and receiving tamoxifen at a dose of 3.3 mg/kg; and *H*. *sabdariffa* + DMBA = Animals receiving *H*. *sabdariffa* calyxes extract at doses of 125, 250, and 500 mg/kg. All animals except those in the normal group (NOR) were exposed to a single dose of DMBA (50 mg/kg; *s*.*c*.). #*p* < 0.05 and ##*p* < 0.01 compared to the normal group (NOR); *∗p* < 0.05 compared to the DMBA group.

#### 3.3.2. Effect on Hematological Parameters

Table 5 presents the results on the hematological parameters. A significant increase in the number of monocytes (*p* < 0.001) and white blood cells (*p* < 0.01) was observed in the DMBA group compared to the normal group. However, a significant decrease in the number of lymphocytes (*p* < 0.001), hemoglobin (*p* < 0.01), and MCHC (*p* < 0.01) was noticed in the DMBA rats compared normal ones. Tamoxifen induced a significant increase in the number of lymphocytes (*p* < 0.01), granulocytes (*p* < 0.01) and MCHC (*p* < 0.05) while it significantly decreased the number of white blood cells (*p* < 0.05) and monocytes (*p* < 0.001) compared to the DMBA group. Animals treated with *H*. *sabdariffa* extract at 125 mg/kg significantly increased the number of granulocytes (*p* < 0.001) and decreased the number of monocytes (*p* < 0.001) when compared with DMBA rats. The *H*. *sabdariffa* extract at 500 mg/kg significantly increased the number of granulocytes (*p* < 0.01) and lymphocytes (*p* < 0.01) while it decreased the number of red blood cells (*p* < 0.05) and monocytes (*p* < 0.05) compared to the DMBA group.

NOR = Animals serving as normal control and receiving distilled water (vehicle); DMBA = Animals serving as negative control and receiving distilled water (vehicle); TAMOX + DMBA = Animals serving as positive control and receiving tamoxifen at a dose of 3.3 mg/kg; and *H*. *sabdariffa* + DMBA = Animals receiving *H*. *sabdariffa* calyxes extract at doses of 125, 250, and 500 mg/kg. All animals except those in the normal group (NOR) were exposed to a single dose of DMBA (50 mg/kg; *s*.*c*.). #*p* < 0.05, ##*p* < 0.01; ###*p* < 0.001 compared to the NOR group; ^*∗*^*p* < 0.05, ^*∗∗*^*p* < 0.01, ^*∗∗∗*^*p* < 0.001 compared to the DMBA group.

#### 3.3.3. Effects on the Microarchitecture of Some Organs

**Figure 4:**
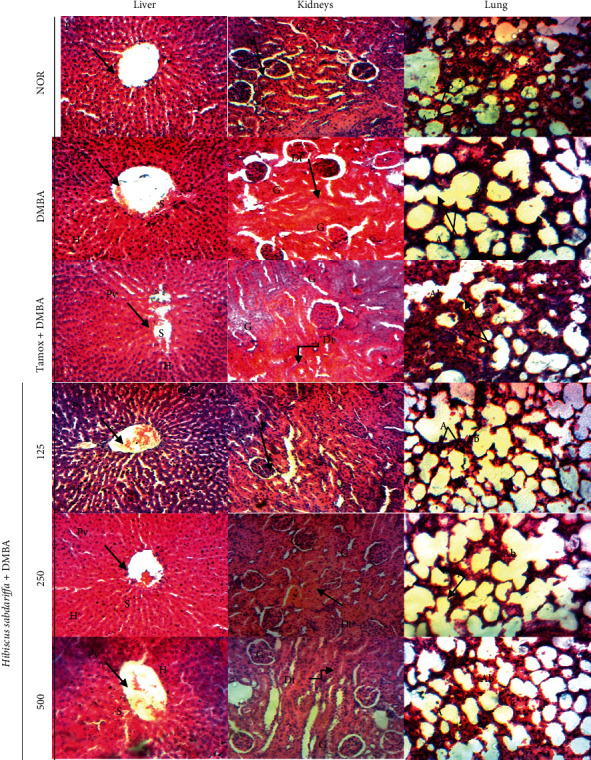
Photomicrographs (HE × 400) showing sections of the liver, kidneys, and lungs after 21 weeks of treatment. NOR = Animals serving as normal control and receiving distilled water (vehicle); DMBA = Animals serving as negative control and receiving distilled water (vehicle); TAMOX + DMBA = Animals serving as positive control and receiving tamoxifen at a dose of 3.3 mg/kg; and (H) sabdariffa + DMBA = Animals receiving (H) sabdariffa calyxes extract at doses of 125, 250, and 500 mg/kg. All animals except those in the normal group (NOR) were exposed to a single dose of DMBA (50 mg/kg; s.c.). Vp = portal vein; *H* = hepatocyte; *S* = sinusoid; *A* = alveolus; Sa = alveolar sac; *G* = glomerulus; Dt = distal tube; Ne = neuron; Co = cortex.

Many organs can be a secondary focus for tumors. The liver, lungs, and brain have been identified as the main sites of breast cancer metastasis in women. In addition, the liver, lungs, and kidneys are the primary organs of exposure to toxic substances. After 21 weeks of treatment, no signs of metastasis and toxicity were observed on the histoarchitecture of the lungs, liver, or kidneys (Figure 4).

## 4. Discussion

Breast cancer is the most common cause of cancer deaths in women [3]. The death of the normal rat observed could be attributed to an accident, since its autopsy showed no macroscopic or histomorphological abnormalities of the organs examined. The high number of deaths observed in DMBA rats could be attributed to its toxicity. Indeed, studies have shown that DMBA induces hemorrhagic lesions (apoplexy) or immunotoxicity in lymphoid organs (spleen, thymus, and bone marrow) [25,26] via its genotoxic metabolite (3,4-dihydrodiol-1,2-epoxide) and the formation of mutagenic-free radicals such as superoxide anion, hydroxyl radical, and peroxides [27]. The low death rate observed in rats treated with tamoxifen is thought to be due to the protective effect of this later against breast cancer [28,29]. Indeed, tamoxifen is a selective estrogen receptor modulator known to be estrogenic on the endometrium and antiestrogenic in the breast [28]. Since estrogen is a promoter of estrogen-dependent breast cancers [30], the antiestrogenic substance inhibits estrogen-dependent cancer growth. The low death rates observed in rats treated with *H sabdariffa* at 125 and 250 mg/kg BW suggest a protective effect against DMBA toxicity. The analgesic property of *H*. *sabdariffa* extract through the inhibition of the release of painful substances such as prostaglandins and bradykinin could help in this issue [31].

Eighty-six percent (86%) of the DMBA-exposed rats developed tumors. Indeed, the environmental carcinogen DMBA-induced breast cancer in rats is one of the most widely used models. However, breast tumors obtained after DMBA exposition are biochemically, immunologically, and histologically similar to that of women [32]. In addition, polycyclic aromatic hydrocarbon (PAHs) from tobacco, grilling, and the incomplete combustion of fossil fuels is one of the most common organic environmental pollutant [33]. The *H*. *sabdariffa* extract at doses of 125 and 250 mg/kg BW as well as tamoxifen significantly inhibited tumor burden compared to the DMBA group. In addition, a significant decrease (*p* < 0.001) in the tumor volume was observed at the dose of 125 mg/kg BW. In line with the later, the concentration of CA15-3 was significantly decreased tested doses. CA 15-3 is the preferred marker for the prognosis of breast cancer as it correlates with the progression, regression, or stability of the disease in patients [34]. In cancer cells, the mucin-1 protein, a transmembrane protein found on the surface of the plasma membranes of epithelial cells in an organism, is overexpressed more particularly breast cancer cells [35]. The CA 15-3 assay, which measures the soluble forms of this protein in serum, has a strong clinical prognosis value [36]. Some authors have also demonstrated that its serum level can slightly vary with menstrual cycle, which could explain the differences observed between the volume of tumors and CA 15-3 levels in this study [37]. The above results suggest a protective effect of *H*. *sabdariffa* extract on breast carcinogenesis, probably due to its ability to scavenge mutagenic-free radicals produced by DMBA. In line with published data, polyphenols and flavonoids, the main bioactive agents in *H*. *sabdariffa* [13], have also been found in high amount in this study. Indeed, some polyphenols such as delphinidin-3-sambubioside, quecertin, and protocatechuic acid from *H*. *sabdariffa* are known to be cytotoxic toward several cancer cells, to arrest cell cycle and trigger apoptosis [13,14,38]. Our data are supported by Shahnaz et al. [39] who demonstrated that *H*. *sabdariffa* aqueous extract inhibited the growth of estrogen-dependent MCF-7 cells through the intrinsic pathway of apoptosis.

Several studies have demonstrated the role of oxidative stress in the carcinogenesis of the human breast [40]. DMBA is well known to initiate part of its tumorigenesis through ROS production. Extensive research has shown that *H*. *sabdariffa* calyxes possess antioxidant properties, thanks to its richness in vitamins, polyphenols, flavonoids, and alkaloids [41]. Furthermore, Anokwuru et al. [42] demonstrated that the antioxidant activity of *H*. *sabdariffa* extract is mostly attributed to phenols. Phenols and flavonoids were found in high amounts in the *H*. *sabdariffa* calyxes extract in this study. There was an increase in SOD activity doses of *H*. *sabdariffa* extract tested and a reduction of the MDA level at the dose of 125 mg/kg. These results were in accordance with the study of Shuruti et al. [40] who also investigated the effect of *H*. *sabdariffa* extract on SOD activity and MDA levels in rats. Indeed, SOD is a metalloprotein which represents one of the first enzymatic lines of defense against oxidative stress by ensuring the elimination of the superoxide anion by a disproportionate reaction [43]. On the other hand, MDA is one of the derivatives of lipid peroxidation and a biomarker of oxidative stress [44]. The antioxidant activity of *H*. *sabdariffa* confirmed in this work would contribute to its chemopreventive activity on breast carcinogenesis.

This study evaluated the relative organ weight, which is an indicator of the possible adverse effects of drugs and toxic compounds [45]. The significant increase in lung wet weight at the dose of 125 mg/kg BW would be attributable to an intergroup variation insofar as no increase was observed in the other treated groups. These results are in accordance with those of Prommetta et al. [46] who demonstrated that *H*. *sabdariffa* calyxes extract does not cause any hepatic damage and does not affect its enzymatic content. Regarding the hematological parameters, the significant decrease (*p* < 0.01) in the hemoglobin level and the significant increase in levels of white blood cells and monocytes in DMBA rats compared to normal rats confirmed the hematotoxicity of this carcinogen. Indeed, the hematopoietic system is one of the most sensitive targets for xenobiotics and therefore constitutes an important indicator of physiological and pathophysiological status [47]. Although not significant, *H*. *sabdariffa* extract increased the number of red blood cells and blood platelets at doses 125 and 500 mg BW. According to Ologundudu et al. [48], the anthocyanins from *H*. *Sabdariffa* calyxes have protective effects against the hemolytic effects induced by carcinogens on blood cells.

## 5. Conclusion

*Hibiscus sabdariffa* calyxes extract reduced animal death, tumor incidence, tumor burden and volume, as well as the level of the breast cancer biomarker CA 15-3. It protected rats against diffuse breast neoplasia induced by DMBA. In addition, this extract showed antioxidant effects characterized by a decrease in the MDA level and a significant increase in the activity of SOD. Taken altogether, these results suggest that the decoction of *H*. *sabdariffa* has antibreast tumor and antioxidant properties in rats. However, the optimal effects were obtained at the smallest dose (125 mg/kg), which must be taken into consideration when preventing cancer.

## Figures and Tables

**Table 1:** Phytochemical constituents of *H*. *sabdariffa* calyxes extract.

	Items	Concentration in *H*. *sabdariffa calyxes extract*
1	Total proteins	17.09 ± 3.07 *µ*g eq BSA/mg DW
2	Polyphenols	288.83 ± 0.5 *µ*g FAE/mg DW
3	Flavonoids	209.76 ± 0.99 *µ*g eq quercetin/mg DW
4	Polysaccharides	29.61 ± 0.03 *μ*g eq glucose/mg DW

DW = dry weight

**Table 2:** Chemoprotective activity of *H*. *sabdariffa* calyxes extract on breast cancerogenesis after 21 weeks post-DMBA exposition.

Item	Number of rats with tumors/group	Incidence of tumors (%)	Tumors burden (g)	% Inhibition of tumor burden
**NOR**	0/8	—	—	—
**DMBA**	7/8	86	913.9 ###	—
**TAMOX** **+** **DMBA**	4/8	50	43.07 ^*∗∗∗*^	95.28
**HS 125** **+** **DMBA**	5/8	63	138.30 ^*∗∗∗*^	84.86
**HS 250** **+** **DMBA**	6/8	75	559.38 ^*∗∗*^	38.78
**HS 500** **+** **DMBA**	5/8	63	702.84	23.09

**Table 3:** Effects of *H*. *sabdariffa* calyxes extract on some markers of oxidative stress after 21 weeks post-DMBA exposition.

	NOR	DMBA	TAMOX + DMBA	*H*. *sabdariffa* + DMBA		
				125	250	500
*Oxidative stress markers*						
(i) MDA (mM/mg of proteins)	4.69 ± 1.81	4.65 ± 0.19	5.96 ± 0.55	2.47 ± 0.50^∗^	3.92 ± 0.63	3.65 ± 0.93
(ii) SOD (unit/mg of proteins)	97.60 ± 0.11	97.33 ± 0.30	99.53 ± 0.17^∗∗^	99.33 ± 0.20^∗∗^	99.45 ± 0.10^∗∗^	99.33 ± 0.20^∗∗^
(iii) Catalase (mM de H_2_O_2_/min/mg of proteins)	0.27 ± 0.0	0.41 ± 0.02^###^	0.27 ± 0.01^∗∗∗^	0.28 ± 0.01^∗∗∗^	0.36 ± 0.01^∗∗^	0.36 ± 0.01^∗^
(iv) Nitric oxide (NO)	51.42 ± 0.56	51.59 ± 0.60	56.84 ± 0.24^∗∗∗^	44.67 ± 5.27	57.51 ± 0.22	56.80 ± 0.17

**Table 4:** Effects of *H*. *sabdariffa* calyxes extract on relative organs weight after 21 week post-DMBA exposition.

Organs	NOR	DMBA	TAMOX + DMBA	*H*. *sabdariffa* + DMBA		
				125	250	500
Uterine	2517.0 ± 312.06	1867.47 ± 131.61	1032.97 ± 199.50##	1811.10 ± 197.86	2204.23 ± 386.86	1691.15 ± 141.83
Liver	29697.76 ± 1569.34	42407.24 ± 4253.22##	33985.34 ± 2625.25	29806.26 ± 1225.70	31352.18 ± 1537.51	36822.72 ± 3116.72
Lungs	7887.11 ± 344.40	8149.09 ± 1116.20	8611.82 ± 657.17	7671.48 ± 282.42*∗*	7129.66 ± 387.71	7903.38 ± 532.89
Spleen	4414.18 ± 447.39	7697.48 ± 1567.35#	4024.03 ± 439.86	4329.94 ± 481.87	4773.63 ± 841.83	4777.12 ± 470.16
Adrenals	270.08 ± 31.58	283.03 ± 54.30	345.58 ± 33.04	266.61 ± 19.90	338.28 ± 63.88	340.04 ± 43.08
Kidneys	5273.57 ± 390.12	7332.25 ± 494.29#	6376.41 ± 695.44	5897.14 ± 273.38	5393.40 ± 219.80	6052.97 ± 697.75
Femur	2586.22 ± 159.75	3572.48 ± 246.80#	3743.98 ± 354.19##	2475.88 ± 104.93	2213.30 ± 147.42	2984.02 ± 170.64
Brain	7925.13 ± 533.72	10437.82 ± 785.13#	10563.13 ± 994.40#	8883.24 ± 360.96	8178.20 ± 380.03	9120.69 ± 512.67
Ovaries	560.04 ± 92.23	528.24 ± 33.72	474.80 ± 71.28	656.40 ± 80.77	548.23 ± 92.59	505.45 ± 146.32

**Table 5:** Effects of H. sabdariffa calyxes extract on some hematological parameters after 21 weeks post-DMBA exposition.

Parameters	NOR	DMBA	TAMOX + DMBA	*H*. *sabdariffa* + DMBA		
				125	250	500
WBC (×10^3^ *µ*L^−1^)	10.60 ± 1.09	21.56 ± 1.96##	12.00 ± 1.91^*∗*^	14.99 ± 2.61	25.14 ± 3.45	12.00 ± 1.78^*∗*^
Lymphocytes (%)	78.04 ± 2.56	43.81 ± 6.55###	70.02 ± 5.26^*∗∗*^	58.5 ± 6.98	33.24 ± 2.05	68.22 ± 4.70^*∗∗*^
Monocytes (%)	19.21 ± 2.71	47.14 ± 5.45###	16.12 ± 4.30^*∗∗∗*^	8.53 ± 1.78^*∗∗∗*^	37.86 ± 6.37	26.81 ± 4.38^*∗*^
Granulocytes (%)	0.86 ± 0.17	0.34 ± 0.04	1.31 ± 0.13^*∗∗*^	1.5 ± 0.19^*∗∗∗*^	0.53 ± 0.16	1.3 ± 0.23^*∗∗*^
RBC (×10^3^ *µ*L^−1^)	8.16 ± 0.69	5.82 ± 0.50	7.58 ± 0.34	7.85 ± 1.64	3.96 ± 0.77	6.41 ± 0.99
Hematocrite (%)	46.1 ± 3.18	30.75 ± 3.68	38.86 ± 4.19	36.7 ± 3.94	26.26 ± 4.73	36.08 ± 3.71
MCV (fL)	57.41 ± 2.33	59.96 ± 2.610	56.28 ± 0.95	65.41 ± 3.01	68.45 ± 4.18	59.9 ± 1.50
Platelets(×10^3^ *µ*L^−1^)	574.57 ± 34.38	461.8 ± 50.09	614.93 ± 18.46	423.4 ± 122.32	449.33 ± 77.48	479.8 ± 56.15
MCH (pg)	18.62 ± 1.59	16.06 ± 0.58	17.51 ± 0.36	23.61 ± 7.23	16.61 ± 0.48	17.86 ± 1.01
Hemoglobin (g/dL)	14.64 ± 0.71	9.31 ± 1.13##	12.06 ± 1.31	10.68 ± 0.93	5.5 ± 0.96	11.03 ± 1.55
MCHC (g/dL)	32.17 ± 1.25	27.23 ± 1.15##	31.18 ± 0.67^*∗*^	27.18 ± 0.93	24.61 ± 0.92	27.31 ± 1.03
IG (%)	0.12 ± 0.04	0.6 ± 0.23	0.32 ± 0.11	1.03 ± 0.30	0.9 ± 0.27	0.15 ± 0.06

NOR = Animals serving as normal control and receiving distilled water (vehicle); DMBA = Animals serving as negative control and receiving distilled water (vehicle); TAMOX + DMBA = Animals serving as positive control and receiving tamoxifen at a dose of 3.3 mg/kg; and *H*. *sabdariffa* + DMBA = Animals receiving *H*. *sabdariffa* calyxes extract at doses of 125, 250, and 500 mg/kg. All animals except those in the normal group (NOR) were exposed to a single dose of DMBA (50 mg/kg; *s*.*c*.). ^#^*p* < 0.05, ^##^*p* < 0.01; ^###^*p* < 0.001 compared to the NOR group; ^*∗*^*p* < 0.05, ^*∗∗*^*p* < 0.01, ^*∗∗∗*^*p* < 0.001 compared to the DMBA group.

## Data Availability

The data and materials used in this study are available upon request from the authors.

## Abbreviations

BW: Body weight
BSA: Bovine serum albumin
CA 15-3: *Cancer* antigen 15-3
DMBA: 7,12 dimethylbenz(a)anthracene
DMSO: Dimethylsulfoxide
EDTA: Ethylenediaminetetraacetic
ELISA: enzyme-linked immunosorbent assay
ER: Estrogen receptor
FA: Formic acid
Hb: Hemoglobin
Ht:hematocrit:MDA: hematocrit:MDA:Malondialdehyde
MCH: mean corpuscular hemoglobin
MCHC: Mean corpuscular hemoglobin concentration
MCV: Mean corpuscular volume
NHC: National Herbarium of Cameroon
NO: Nitric oxide
NO: normal control
PAH: Polycyclic aromatic hydrocarbon
RBC: red blood cell
ROS: Reactive oxygen species
SEM: Standard error of mean
SOD: Superoxide dismutase
SERM: Selective estrogen receptor modulator
TAMOX: Tamoxifen.
